# P-MAPA immunotherapy potentiates the effect of cisplatin on serous ovarian carcinoma through targeting TLR4 signaling

**DOI:** 10.1186/s13048-018-0380-5

**Published:** 2018-01-17

**Authors:** Luiz Gustavo de Almeida Chuffa, Grazielle de Moura Ferreira, Luiz Antonio Lupi, Iseu da Silva Nunes, Wagner José Fávaro

**Affiliations:** 1 Department of Anatomy, São Paulo State University (Unesp), Institute of Biosciences, Rubião Júnior, s/n, P.O Box: 18618-970, Botucatu, SP 510 Brazil; 2 Farmabrasilis R&D Division, Campinas, SP Brazil; 3 Department of Structural and Functional Biology, Laboratory of Urogenital Carcinogenesis and Immunotherapy, UNICAMP - University of Campinas, Campinas, SP Brazil

**Keywords:** Ovarian cancer, P-MAPA, Cisplatin, TLR2, TLR4, NF-kB

## Abstract

**Background:**

Toll-like receptors (TLRs) are transmembrane proteins expressed on the surface of ovarian cancer (OC) and immune cells. Identifying the specific roles of the TLR-mediated signaling pathways in OC cells is important to guide new treatments. Because immunotherapies have emerged as the adjuvant treatment for patients with OC, we investigated the effect of a promising immunotherapeutic strategy based on protein aggregate magnesium-ammonium phospholinoleate-palmitoleate anhydride (P-MAPA) combined with cisplatin (CIS) on the TLR2 and TLR4 signaling pathways via myeloid differentiation factor 88 (MyD88) and TLR-associated activator of interferon (TRIF) in an in vivo model of OC.

**Methods:**

Tumors were chemically induced by a single injection of 100 μg of 7,12-dimethylbenz(a)anthracene (DMBA) directly under the left ovarian bursa in Fischer 344 rats. After the rats developed serous papillary OC, they were given P-MAPA, CIS or the combination P-MAPA+CIS as therapies. To understand the effects of the treatments, we assessed the tumor size, histopathology, and the TLR2- and TLR4-mediated inflammatory responses.

**Results:**

Although CIS therapy was more effective than P-MAPA in reducing the tumor size, P-MAPA immunotherapy significantly increased the expressions of TLR2 and TLR4. More importantly, the combination of P-MAPA with CIS showed a greater survival rate compared to CIS alone, and exhibited a significant reduction in tumor volume compared to P-MAPA alone. The combination therapy also promoted the increase in the levels of the following OC-related proteins: TLR4, MyD88, TRIF, inhibitor of phosphorylated NF-kB alpha (p-IkBα), and nuclear factor kappa B (NF-kB p65) in both cytoplasmic and nuclear sites. While P-MAPA had no apparent effect on tumor necrosis factor alpha (TNF-α) and interleukin (IL)-6, it seems to increase interferon-γ (IFN-γ), which may induce the Thelper (Th1)-mediated immune response.

**Conclusion:**

Collectively, our results suggest that P-MAPA immunotherapy combined with cisplatin could be considered an important therapeutic strategy against OC cells based on signaling pathways activated by TLR4.

## Background

Ovarian cancer (OC) is the most lethal of all gynecological cancers and predicts a poor outcome due to late diagnosis (< 50% in a 5-year relative survival rate) [1]. Most patients present with no apparent signs or symptoms in the early stage of OC, and no effective screening protocols are currently available [2]. OC is classified into different subtypes and often arises from lesions involving the surface epithelium and its inclusion cysts [1, 2]; however, most high-grade serous carcinomas (> 70%) are derived from the epithelium in the fimbriated part of the fallopian tube. Notably, the development of chemoresistance is the key factor that hinders the sensitivity of OC cells to long-term treatment [2], and cisplatin/paclitaxel resistance has been shown to induce recurrent OC and metastasis [3].

TLRs are active molecules located on the cell surface that can trigger a cytoplasmic signaling pathway to activate nuclear factors (e.g. NF-kB, IRF) leading to cell proliferation, resistance to drugs, and recruitment of immune cells to the tumor site [4]. The TLR system constitutes a double-edge sword that needs specific investigation in the setting of neoplastic disease. Particularly, TLR4 has been recognized as a key element in promoting the immune evasion of many cancer cells, including OC cells [5–7]. Conversely, TLR signaling was shown to effectively reduce the proliferative capacity of cancer cells [7]. TLRs often act through MyD88-dependent pathway with early activation of NF-kB, an important transcription factor that is responsible for the expression of proinflammatory molecules, such as TNF-α, IL-6, IL-8, and others. These interleukins are associated with inflammation, cell proliferation, adhesion, migration, apoptosis, and angiogenesis [8, 9]. Alternatively, TLRs can activate MyD88-independent pathway via TRIF signaling, resulting in late NF-kB activation and induction of IRF3, which increases the level of IFN [10].

Because TLRs, especially TLR2 and TLR4, are implicated in the pathogenesis of OC [5, 11], compounds displaying either agonist or antagonist activities may represent a potential immunotherapeutic opportunity for cancer treatment, whether used alone or in combination with other therapies. In this immunoadjuvant scenario, the protein aggregate magnesium-ammonium phospholinoleate-palmitoleate anhydride (P-MAPA; developed by Farmabrasilis, Campinas, SP, Brazil) has been identified as an important candidate for cancer therapy. P-MAPA is a biological immunomodulator obtained after fermentation of *Aspergillus oryzae* that exhibits considerable antitumor effects in different experimental models of cancer [12–15]. Recent approaches into the understanding of its mechanism of action showed that P-MAPA efficiently modulated TLR2 and TLR4 in both cancer and infectious diseases, besides stimulating T cells (TCD4+ and TCD8+ cells) and natural killer (NK) cell responses [12–15].

Cis-Diamminedichloroplatinum (cisplatin or “CIS”) is a potent antitumor agent, which is active against a variety of cancers, including OC [16, 17]. Notably, the antineoplastic properties of CIS have been fully ascribed to its radiosensitizing effect and immunomodulatory ability [18]. In OC cells, apoptosis is triggered by CIS treatment and chemoresistance is associated to a reduced susceptibility to undergo cell death [19]. CIS is also capable of increasing the expression of TLRs and activate MAPK by inducing the phosphorylation of IkB. The CIS-induced expression of TLRs, especially TLR2 and TLR4, is reduced in the presence of MAPK and NF-kB inhibitors [20]. Although the mechanisms of conventional platinum chemotherapy are well established for OC, further studies with new adjuvant drugs may add therapeutic value upon activation of TLRs within the tumor microenvironment.

To understand the effectiveness of these compounds, the present study was conducted to verify the immunomodulatory effect of P-MAPA associated or not to CIS on the TLR2- and TLR4-mediated signaling pathways in an animal model of OC. This model exhibits the appropriate pathophysiological and molecular features that are required to test new functional chemotherapeutic substances.

## Methods

### Animals and treatments

Forty adult female Fischer 344 rats (60-day-old, weighing ±200 g), were obtained from the Multidisciplinary Center for Biological Investigation (CEMIB) at University of Campinas (UNICAMP). Animals were individually housed in polypropylene cages with laboratory-grade pine shavings as bedding and kept in a climatized room under controlled temperature at 22 ± 1 °C with a 12/12 h light/dark cycle, lights switched on at 6:00 a.m. During the procedures, standard rodent food (3074 SIF, Purina Ltda., Campinas, SP, Brazil) and filtered tap water were provided ad libitum. The animals were divided into four groups (*n* = 10/group): OC: composed of rats that were induced with 7,12-dimethylbenz(a)anthracene (DMBA) and received vehicle only; OC + P-MAPA: composed of rats that were induced with DMBA and received P-MAPA as therapy; OC + CIS: composed of rats that were induced with DMBA and received cisplatin as therapy; OC + P-MAPA+CIS: composed of rats that were induced with DMBA and received simultaneous doses of P-MAPA and cisplatin as treatment. This chemically-induced rat model of OC is appropriate for studying the structural and functional components of the tumor microenvironment, including the immune system [21, 22].

**Fig. 1:**
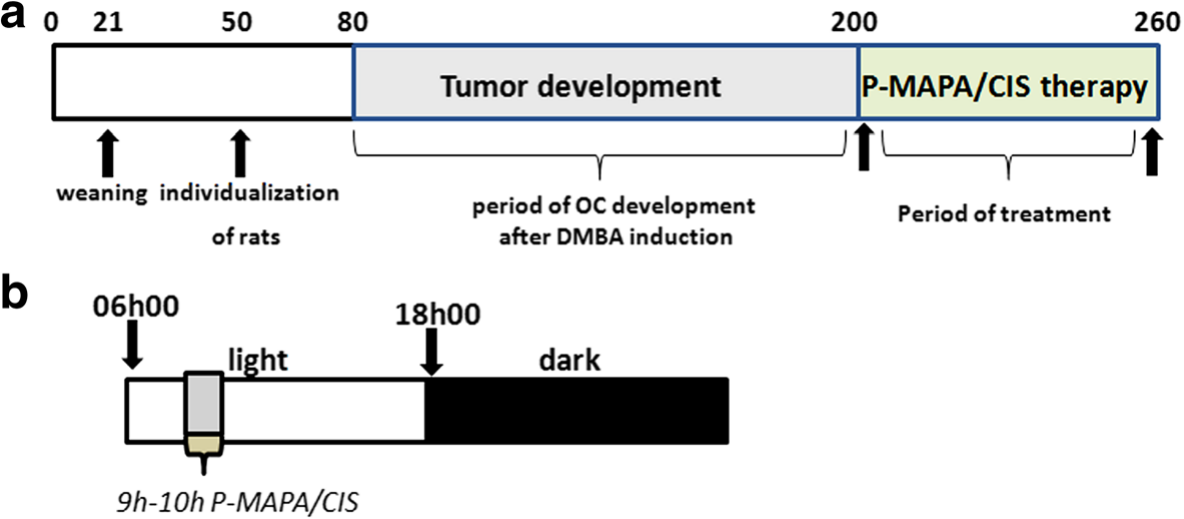
Chronological scheme of the experimental design. **a** Timeline for OC development and period of P-MAPA and CIS therapy (days). **b** The schematic protocol used for P-MAPA and CIS administration (twice a week: 9–10 h)

After OC was developed (200-day-old), the animals designated to receive P-MAPA were administered i.p. doses of 5 mg/kg b.w. dissolved in 0.20 mL of 0.9% (*v*/v) saline solution (vehicle) at a final concentration of 5 mg/mL [13]. The animals treated with cisplatin received two doses of 5 mg/kg, via i.p., twice a week (on Monday and Thursday), dissolved in 0.20 mL of the vehicle. The combination therapy was administered twice a week via i.p. (5 mg/kg P-MAPA and 5 mg/kg CIS). Control animals received injections of the vehicle in the same procedures of treated animals. The injections were administered in the morning (between 09:00 and 10:00 h, Fig. 1a and b), twice a week for 8 consecutive weeks. After the experimental period, all animals were euthanized by decapitation (during early morning at 8:00 a.m.) for sample collection.

### Ethics statement

To minimize any kind of discomfort and pain during experimentation, the rats were anesthetized with ketamine and xylazine so that they were rendered unconscious. Euthanasia was performed in accordance with the Canadian Council on Animal Experimentation. This experimental protocol was previously approved by the Ethical Committee of the Institute of Bioscience/UNESP, Campus of Botucatu, SP, Brazil (Permit Number: 950).

### Procedures for OC induction

After defining the experimental groups, all the animals (*n* = 40) were anesthetized using 10% ketamine (60 mg/kg, i.p., Vibra® Roseira, SP, Brazil) and 2% xylazine (5 mg/kg, i.p., Vibra® Roseira, SP, Brazil) during the estrous stage. The left flank of the animals was incised through the skin and abdominal wall to access the ovaries. The left ovaries were injected direct under the ovarian bursa with a dose of 100 μg of DMBA (Sigma Chemical Co, St. Louis, MO) dissolved in 10 μL of sesame oil (vehicle) [23–25]. Right ovaries were injected with the vehicle only (sham-surgery). After the ovaries were moved back to the abdominal cavity, muscle layers and skin were tightly closed using a 3–0 silk suture, and prophylactic treatment with antibiotic benzylpenicillin potassium was given to the animals. Over the next 120 days of development, tumors were monitored by ultrasound to predict the volume and total size.

### OC histopathology

After euthanasia, the animals were necropsied and studied for general systemic abnormalities. During necropsy, the internal reproductive organs, and those related to body metabolism were carefully dissected. Animals displaying evident OC had a portion of a large tumor removed, rapidly frozen, and stored at − 80°C. For the histopathological analyses, serous OC was fixed in 10% buffered formalin followed by dehydration, clearing, and paraffin embedding. The tissue was serially sectioned at 5-μm thickness, and every 10th tissue section was stained with hematoxylin and eosin. Histopathological data were analyzed and reported by a pathologist.

### Immunohistochemistry

Tissue sections of papillary OCs were deparaffinized and microwaved at 700 W using 0.01-M sodium citrate buffer (pH 6.0). After antigen retrieval, the peroxidase activity was blocked, and OC tissues were incubated with 3% BSA for 1 h. Then, OC sections were incubated overnight with primary antibodies (Abcam, Cambridge, UK): rabbit polyclonal anti-TLR2 (1:100) and mouse monoclonal anti-TLR4 (1:100). After the reactions, tissues were washed 3× and incubated with polymer Anti-Mouse IgG or Anti-Rabbit (DAKO ® CYT) at room temperature (RT) for 1 h. Finally, the slides were reacted with diaminobenzidine (DAB; Sigma, St. Louis, MO), and OC sections counterstained with hematoxylin for 1 min. To eliminate false positive reactions, control slides were obtained without the primary antibody. Acquisition of all images was performed under a microscope (Zeiss Axiophot II, Carl Zeiss, Oberkochen, Germany), considering the intensity of immunoreactivity as absent (0), low (+), moderate (++), or high (+++).

### Western blot analysis

After the experiments, papillary OCs were removed and rapidly frozen in liquid nitrogen and stored at − 80°C. To obtain cytoplasmic and nuclear extracts, OC samples were fractionated using NE-PER extraction reagents (Pierce, Rockford, IL). After protein extraction, 10% (*v*/v) triton X-100 was added to the solution for 2 h. Then, lysates were centrifuged according to the manufacturer’s instructions, and protein quantification was performed using Bradford protein assay. The same amount of protein (50 μg) was added to 1.5× Laemmli buffer and used for SDS-PAGE (Bio-Rad Laboratories, Hercules, CA) at preformed 4–12% acrylamide gradient gels using a Tris-glycine running buffer system (60 mA for 2 h). The proteins were electro-transferred to nitrocellulose membranes, and then blocked with 3% BSA in TBS-T solution (0.05% Tween in TBS) at RT for 1 h. After blocking, proteins were incubated at 4°C overnight with respective primary antibodies (1:500 or 1:1000 in 1% BSA): TLR2, TLR4, MyD88, TRIF, IKK-α, p-IkBα, and NF-kB p65 (Abcam, Cambridge, UK). Subsequently, the non-binding primary antibodies were removed and membranes were incubated for 2 h at RT with specific secondary antibodies (Sigma-Aldrich, St. Louis, MO) diluted 1:10,000 in 1% BSA. After sequential washes in TBS-T solution, positive bands were detected using ECL kit (Thermo Fisher Scientific, MA). Images of blots were calculated from individual blots of 5 rats/group using Image J software and were represented as the mean optical density (band intensity-pixels). β-actin for cytosolic fraction and Lamin B1 for nuclear fraction were used as endogenous controls.

### Immunofluorescence assay

The OC tissues were isolated, washed in PBS and fixed in 4% paraformaldehyde for 10 min. For tissue permeabilization, proteinase K was used and nonspecific bindings were blocked with 1% BSA for 1 h. Then OCs were incubated overnight with rabbit polyclonal NF-kB p65 antibody diluted 1/100 (ab7970, Abcam, Cambridge, MA), followed by another incubation with secondary rabbit polyclonal anti-IgG conjugated to FITC (1:200 dilution, Santa Cruz Biotechnology, Inc., CA) for 1 h. For nuclei staining, 4,6-diamidino-2-phenylindole (DAPI; Sigma, St Louis, MO) was added (5 min). Positive staining in OC cells was analyzed using a fluorescence microscope (Zeiss Axiophot II, Oberkochen, Germany) at 40× magnification (excitation filter 590 nm, emission filter 650 nm) and for DAPI staining (excitation filter 365 nm, emission filter 485 nm). Relative fluorescence in merged images was calculated using Image J software.

### ELISA assays

For the quantitative determination of proinflammatory cytokines, levels of IFN-γ (cat. R1F00), IL-6 (cat. R6000B), and TNF-α (cat. RTA00) were achieved using similar amounts of proteins extracted from the OC samples. The commercial pre-coated rat Quantikine® ELISA kits were provided by R&D Systems (Minneapolis, MN).

### Statistical analysis

All data were performed using analysis of variance (ANOVA) and presented as the mean ± standard deviation (SD). Significant results were subjected to *post-hoc* analysis with Tukey tests, and statistical significance level was set to *P* < 0.05 for all analyses. Data were analyzed using *GraphPad Prism 5.0* scientific graphing software (GraphPad Software, San Diego, CA).

## Results

### Incidence of OC and tumor sizes affected by P-MAPA and CIS

**Fig. 2:**
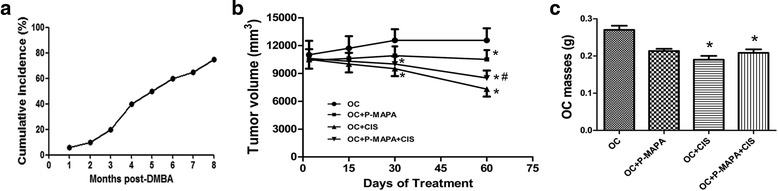
Development of OC and tumor size. **a** Incidence rate (%) of developed OC from 1 to 8 months after DMBA infusion. **b** The tumor volume (mm^3^) was measured throughout the experiment. ^*^ *P* < 0.05 vs. the OC group; # *P* < 0.05 vs. the OC + P-MAPA group. **c** The tumor mass (g) was recorded at the end of treatments. ^*^ *P* < 0.05 vs. the OC group

The OC incidence rate was increased from 4 to 8 months after DMBA injection reaching almost 80% of the total developed neoplasms (Fig. 2a). We initially inoculated 13 animals by group, and at least 10 animals exhibited aggressive carcinoma; no death occurred during tumor development. To confirm the effect of P-MAPA and CIS on this rat model of OC, tumor volume and masses were investigated. Animals treated with CIS alone and the combination of P-MAPA with CIS showed an effective reduction in OC volume after 30 days of treatment (24.4% and 20.3%, respectively, compared with the OC group; Fig. 2b). Most notably, P-MAPA and CIS alone or in combination significantly reduced the tumor volume after 60 days of treatment (16.3%, 41% and 32.2%, respectively, compared with the OC group; Fig. 2b). The combination of P-MAPA with CIS was still more efficient in promoting a reduction in OC volume compared to P-MAPA alone (20% reduced after 60-day treatment). At the end of treatments, CIS- and P-MAPA+CIS-treated groups exhibited a significant decrease in OC masses (30% and 26%, respectively, lower than the OC group; Fig. 2c).

### Anatomopathological analysis of OC, survival rates, and histological features

**Fig. 3:**
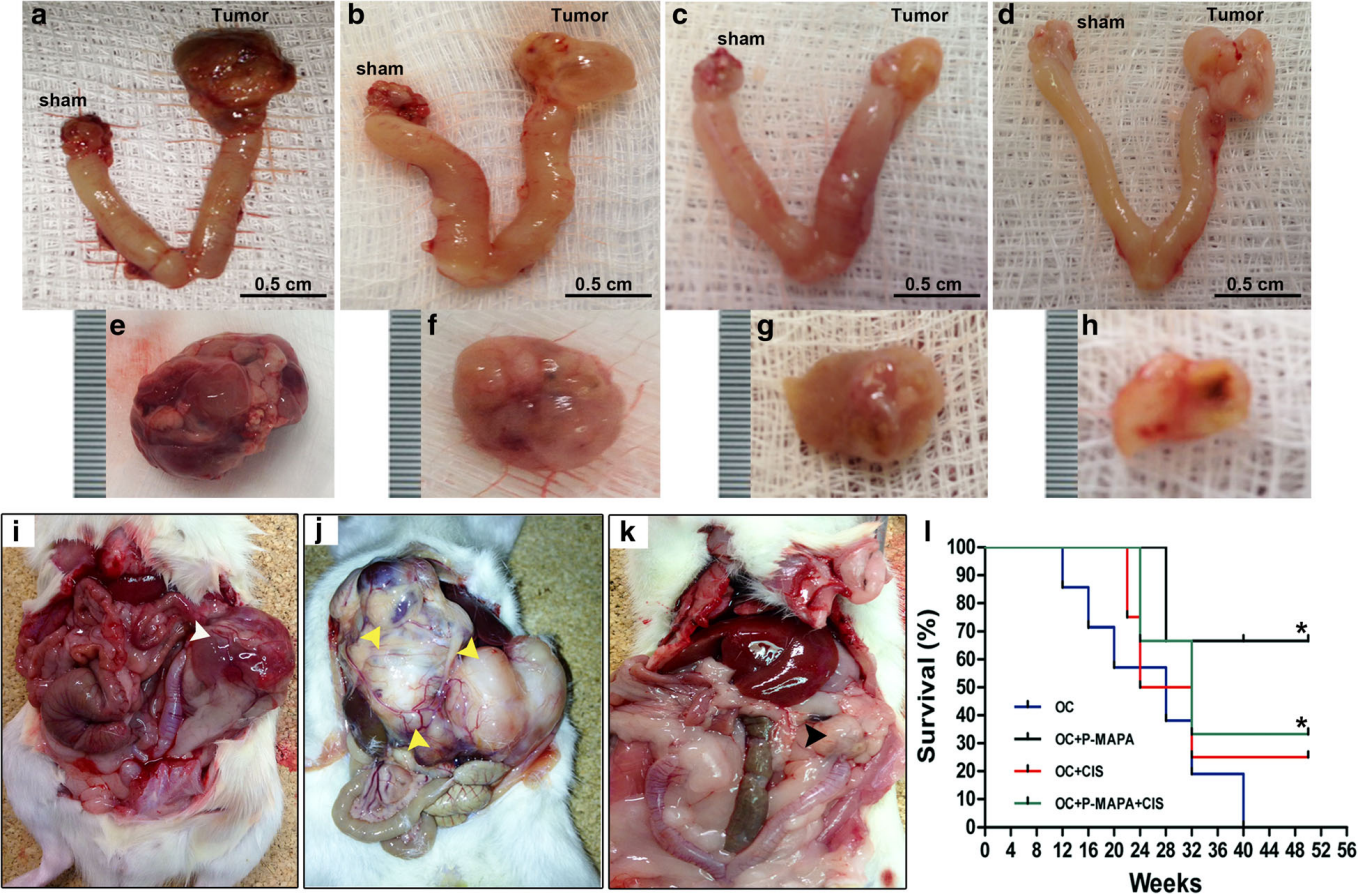
Images of the most common OC after 180 days of DMBA induction. The left ovaries of animals in the OC group presented a solid mass with necrotic spots (**a** and **c**). The left ovary of an animal treated with P-MAPA displayed a large cyst with serous secretions (**b** and **f**). CIS-treated (**c** and **g**) and P-MAPA+CIS-treated (**d** and **h**) animals presented a soft and mobile OC tissue with few adhesions. Right ovaries were sham-operated controls. **i** Abdominal cavity was opened to show an untreated neoplasm (white arrowhead). **j** A number of peritoneal implants (yellow arrowhead) with complex abdominal adhesions appeared in some untreated animals. **k** Left OC (black arrowhead) of a treated animal after the combination of P-MAPA and CIS. **l** Kaplan-Meier Curve showing animal survival rates (%) following the treatments (log-rank test *p* = 0.031; * shows significant differences from the OC group)

**Fig. 4:**
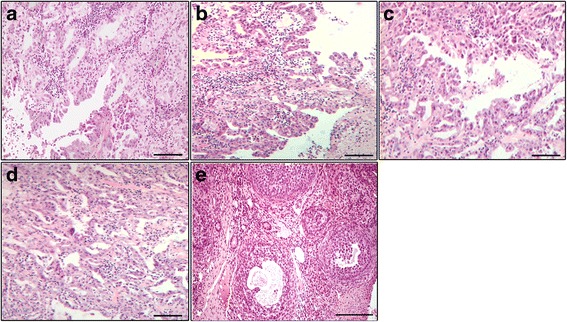
Photomicrographs of the OC. High-grade serous OC showing an exophytic papillary architecture in animals from the OC (**a**), P-MAPA (**b**), CIS (**c**), and P-MAPA+CIS (**d**) groups. Most of these adenocarcinomas exhibited slit-like spaces with high nuclear grade and mild cellular atypia. Sham-operated right ovary with normal follicular development (**e**). Bar = 50 μm; H&E stain

After 180 days of OC induction, the tumors were carefully assessed. Most of the left ovaries from the OC group showed solid mass with scattered areas of necrosis (Fig. 3a, e, i). Furthermore, these animals showed many peritoneal implants followed by abdominal adhesions composed of fibrous bands (Fig. 3j); when untreated, 100% of the control females died at 280 days after DMBA induction (Fig. 3l). In addition, the majority of P-MAPA-treated animals exhibited OC with serous fluid-filled cysts (Fig. 3b, f). Supported by reduced tumor volume and masses, the left ovaries of the animals in the CIS and P-MAPA+CIS groups had great morphological differences evidenced by mobile and soft pale tissue with few adhesions (Fig. 3c, d, g, h). Conversely, the right ovaries (sham-operated animals) appeared largely intact with no macroscopic lesion. Figure 3k shows a non-expansive and controlled OC in an animal that received the combination of P-MAPA and CIS as therapy. To evaluate the prognostic value of the different treatments, we first investigated non-treated animals with OC over several weeks after tumor induction (Fig. 3l). As expected, animals from the OC group revealed an impressive shorter survival rate (280 days after OC acquisition). Surprisingly, treatment with P-MAPA showed the longer survival rate by about 65% (~ 462 days), whereas CIS and P-MAPA+CIS treatments only had an increase in animal survival by about 25% (~ 350 days) and 35% (~ 380 days), respectively, from 32 to 50 weeks after tumor induction (Fig. 3l). After the period of OC development, the majority of tumors were malignant carcinomas classified as high-grade serous papillary (Fig. 4). In general, OCs showed a mixture of papillary and cystic pattern with extensive projections, cellular atypia, and stromal invasion. Furthermore, the presence of scattered mitosis and tumor-infiltrating lymphocytes was obvious, thus, reflecting a potential compartment to be challenged with immunostimulants; although the tumor size was significantly reduced, no histopathological changes were observed after P-MAPA and CIS treatments (Fig. 4a-e).

### Immunotherapy with P-MAPA potentiated the effects of CIS upon TLR4 expression

**Fig. 5:**
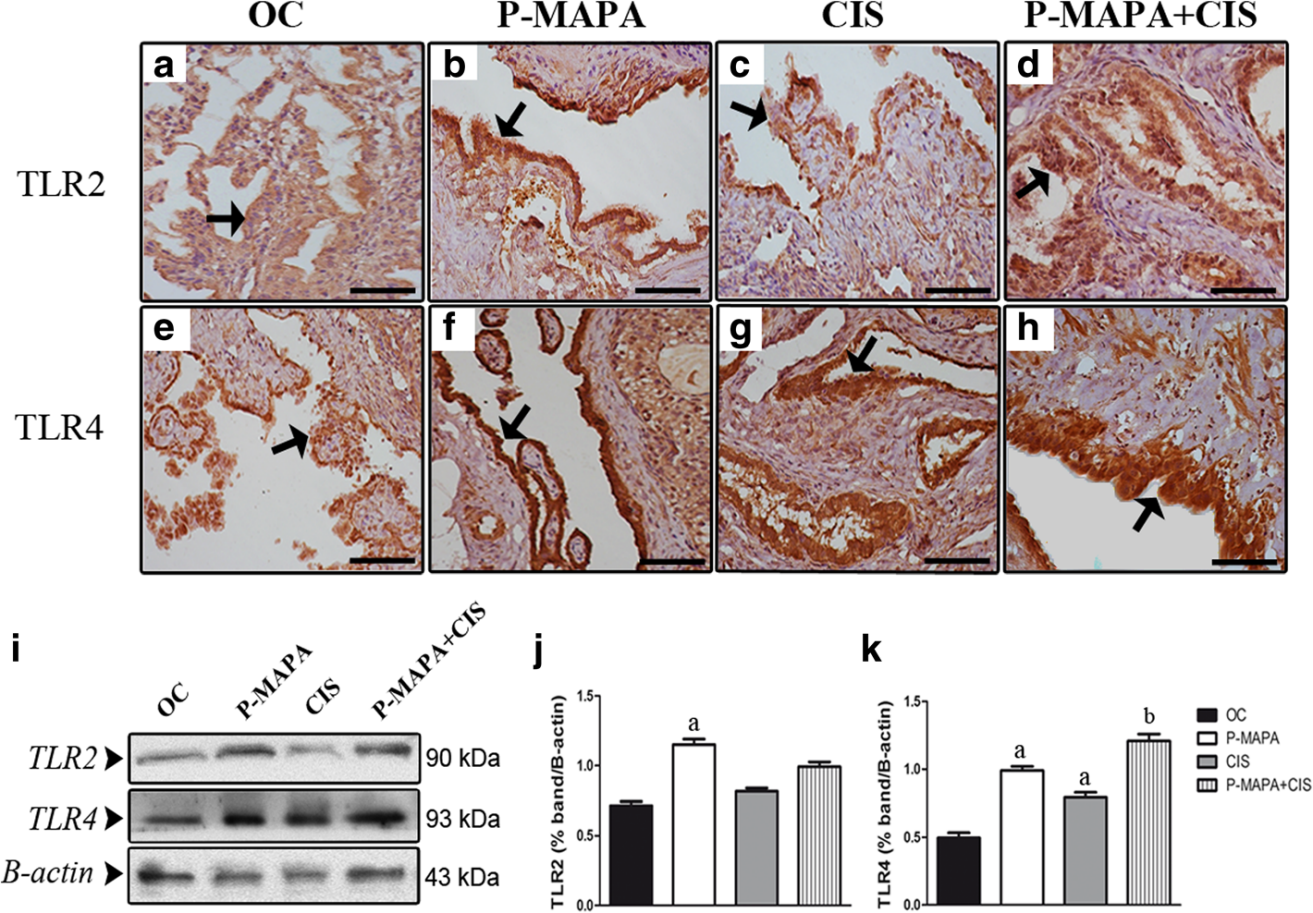
Immunohistochemistry and western blot analysis of TLR2 and TLR4 in serous papillary OC. The immunoreactivity for TLR2 was moderate (arrow) in the P-MAPA (**b**) compared to a weak immunostaining in OC animals (**a**). While CIS therapy (**c**) showed a weak immunoreaction for TLR2, the combination of P-MAPA and CIS exhibited variations from weak to moderate (**d**) (arrows). TLR4 immunoreactivity was intense (arrow) in the P-MAPA (**f**) compared to weak reaction in the OC animals (**e**). In addition, the combination of P-MAPA with CIS promoted an intense immunoreaction to TLR4 (**h**) compared to CIS therapy (**g**) (arrows). Bar = 20 μm. Negative controls were used. **i** Expression profile of the TLR2 and TLR4 in extracts of 50 μg proteins pooled from 5 samples per group. **j**, **k** Individual blots were used for densitometric analysis of the TLR2 and TLR4 levels following normalization to the β-actin. Data are expressed as the mean ± SD. ^a^ *P* < 0.05 vs. OC; ^b^ *P* < 0.05 vs. CIS

In response to P-MAPA immunotherapy, the expressions and immunostaining of TLR2 were enhanced (1.46-fold increase vs. OC) in the cells of serous papillary OCs (Table 1, Fig. 5a, b, i, j). CIS therapy and the combination of P-MAPA with CIS showed no significant effect on the TLR2 levels (*p* > 0.05; Table 1, Fig. 5c, d, i, j). Notably, P-MAPA therapy positively regulated TLR4 in these cells (1.50-fold increase vs. OC; Table 1, Fig. 5e, f, i, k), and CIS treatment also upregulated TLR4 but not in the same intensity as P-MAPA did (1.37-fold increase vs. OC; Table 1, Fig. 5e, g, i, k). Unexpectedly, the combination of P-MAPA with CIS promoted a higher overexpression of TLR4 compared to the therapies alone (1.02- and 1.36-fold increase vs. P-MAPA and CIS groups, respectively; Table 1, Fig. 5h, i, k).

**Table 1:** Immunohistochemical staining intensity in OC cells

Proteins	Therapeutic strategy			
	OC	OC + P-MAPA	OC + CIS	OC + P-MAPA + CIS
*TLR2*	+	++	+	+/++
*TLR4*	+	++/+++	++	++/+++

OC immunostaining was interpreted and scored by two independent pathologists as 0 (absent), + (low), ++ (moderate), or +++ (high). *N* = 5 animals/group. Selected OC was analyzed using five sections per animal

### Upregulation of NF-kB signaling is enhanced by P-MAPA and CIS treatments

**Fig. 6:**
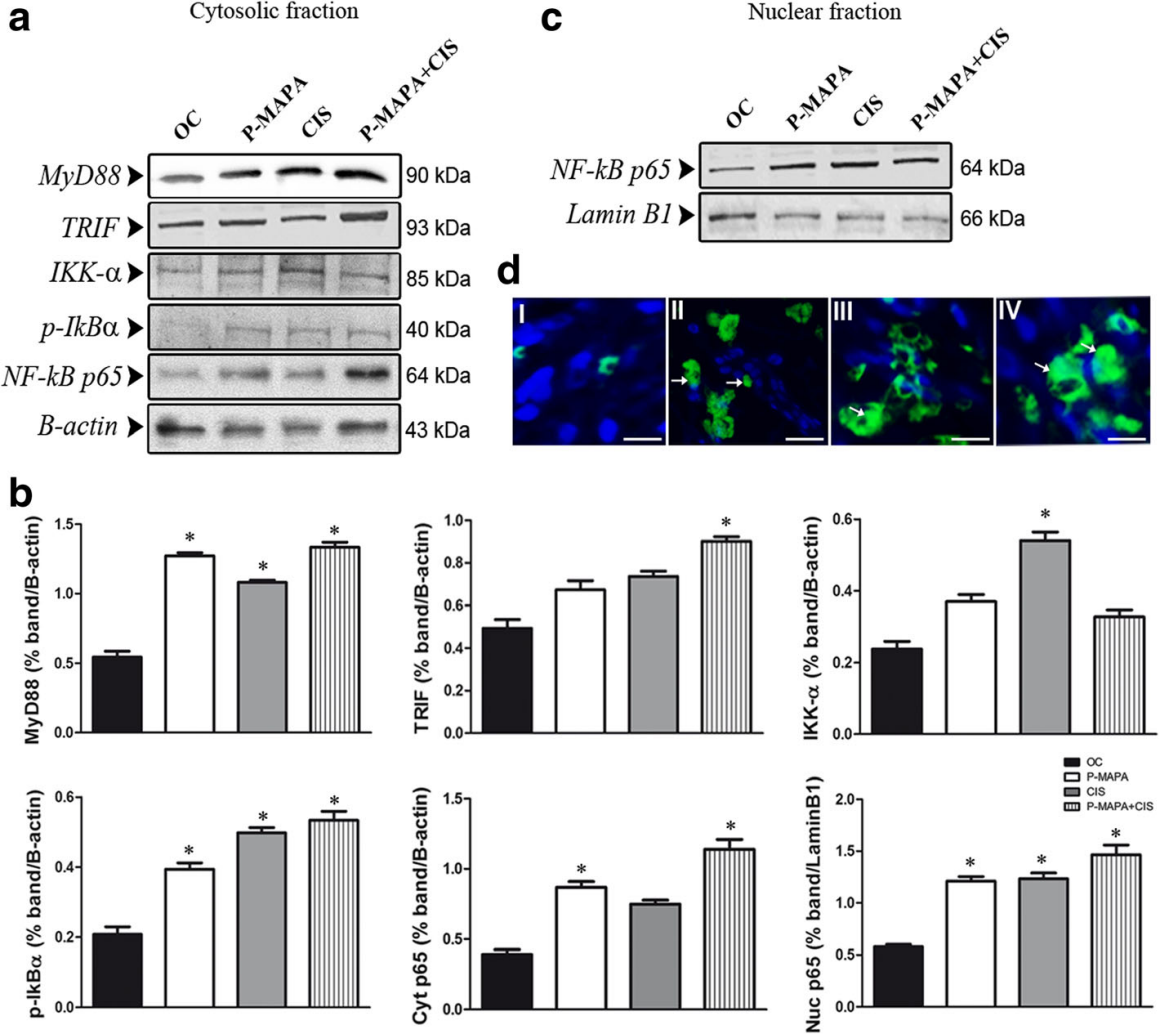
Western blot analysis of MyD88, TRIF, IKK-α, p-IkBα, and NF-kB p65 and fluorescence localization in serous papillary OC. Protein profiles of representative cytosolic (**a**) and nuclear (**c**) fractions (50 μg protein) pooled from 5 samples per group. **b** Individual blots were used for densitometric analysis of the protein levels following normalization to β-actin or Lamin B1. Data are expressed as the mean ± SD. ^*^ *P* < 0.05 vs. the OC group. **d** Merged images of NF-kB p65 immunofluorescence and DAPI nuclear staining in the OC (I), P-MAPA (II), CIS (III), and P-MAPA+CIS (IV) groups (Alexafluor®488, Bar = 10 μm). MyD88: Myeloid differentiation factor 88; TRIF: TLR-associated activator of interferon; IKK-α: inhibitor of NF-kB kinase subunit alpha; p-IkBα: inhibitor of phosphorylated NF-kB subunit alpha; NF-kB p65: NF-kB subunit p65 (RelA)

To investigate the effects of P-MAPA and CIS upon MyD88- or TRIF-dependent signaling pathways, these adaptor molecules were measured in the OC tissues. Interestingly, P-MAPA and CIS alone or even the combination of P-MAPA and CIS promoted an increase in MyD88 levels (1.56-, 1.48-, and 1.58-fold increases, respectively vs. OC; Fig. 6a, b), thereby providing evidence for a MyD88-dependent TLR4-mediated signaling. In addition, the combination of P-MAPA with CIS resulted in the elevation of TRIF levels (1.45-fold increase vs. OC; Fig. 6a, b), being able to further activate downstream molecules via the TRIF-dependent signaling pathway.

The characterization of NF-kB signaling pathway was also evaluated in both cytoplasmic and nuclear extracts of OC following the expressions of IKK-α, p-IkBα, and NF-kB p65 subunit. While IKK-α was only upregulated by CIS treatment (1.57-fold increase vs. OC; Fig. 6a, b), the phosphorylation of IkBα was significantly increased by P-MAPA, CIS, and the combination P-MAPA+CIS (1.45-, 1.55-, and 1.60-fold higher, respectively vs. OC; Fig. 6a, b), thus proving that IKK complex is active in mediating IkB phosphorylation, and consequently, its degradation. Surprisingly, P-MAPA and the combination of P-MAPA with CIS induced the upregulation of cytosolic NF-kB p65 (1.61- and 1.69-fold increases, respectively vs. OC; Fig. 6a, b), and more effectively, P-MAPA, CIS, and P-MAPA+CIS treatments dramatically enhanced nuclear NF-kB p65 (1.49-, 1.50-, and 1.58-fold increases, respectively vs. OC; Fig. 6b, c); particularly, a considerable amount of the NF-kB p65 complex was translocated into the nucleus. Immunofluorescence assay further indicated the localization and expression level of cytoplasmic and nuclear NF-kB p65 in OC cells. P-MAPA immunotherapy resulted in high p65 expression (Fig. 6d I and II; the level of fluorescence increased from 43% ± 0.67 (OC) to 86% ± 11.3 (P-MAPA) as well as CIS therapy (Fig. 6d I and III; the level of fluorescence increased from 43% ± 0.67 (OC) to 102% ± 14.1 (CIS). Lastly, the combination of P-MAPA and CIS promoted the highest p65 immunofluorescence intensity (134% ± 17.1; Fig. 6d IV).

### IFN-γ, TNF-α, and IL-6 levels in OC following P-MAPA and CIS therapy

**Fig. 7:**
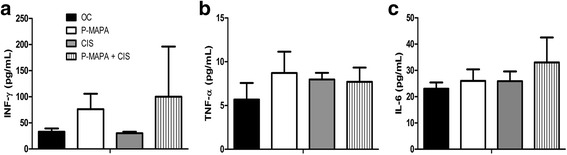
ELISA assays of the IFN-γ, TNF-α and IL-6 levels. Supernatants of OC samples with papillary carcinoma were obtained at the end of treatments. IFN-γ: interferon-gamma; TNF-α: tumor necrosis factor alpha; IL-6: interleukin-6. All data are expressed as the mean ± SD. ANOVA complemented by Tukey test was performed

To verify the effects of therapies on pro-inflammatory cytokines, the representative levels of IFN-γ, TNF-α, and IL-6 were measured in serous OC samples of animals and were reported to be 33 ± 6.1 pg/mL, 5.68 ± 1.88 pg/mL and 23.3 ± 2.38 pg/mL, respectively (Fig. 7a–c). Treatment with P-MAPA and the combination P-MAPA + CIS promoted an increase in the concentration of IFN-γ in OC tissues (1.56- and 1.67-fold increases, respectively vs. the OC group; Fig. 7a). The levels of TNF-α and IL-6 were unchanged in serous OC after P-MAPA and CIS treatments (Fig. 7b and c).

## Discussion

We reported that CIS and P-MAPA+CIS therapies are efficient in reducing OC volume and mass, evidenced by a soft and mobile tissue; in addition, the treatments significantly increased overall animal survival. These results clearly demonstrate the effectiveness of CIS rather than P-MAPA in promoting the reduction of tumor growth. CIS treatment, a first-choice chemotherapeutic agent for treating OC, is associated with tumor growth inhibition in human and animal models [26, 27], and P-MAPA was unable to sensitize OC cells to CIS effects regarding tumor reduction. We also verified that this animal model does not survive more than 280 days after chemical induction of OC, and intriguingly, P-MAPA therapy led to the longer survival rate in these OC-bearing animals. Thus, it can be presumed that P-MAPA might be controlling molecular events linked to OC progression and widespread metastasis.

In non-treated animals, we observed remarkable macroscopic changes in the OCs, including tumors with solid mass and necrosis, abdominal adhesions, and peritoneal implants. The combination of P-MAPA with CIS was much more effective than therapy alone in terms of significant reduction of OC sizes and peritoneal adhesions. Interestingly, a recent work reported that P-MAPA immunotherapy had a promising effect in the histopathological recovery of the bladder cancer state [14]. Regarding OC histopathology, P-MAPA was unable to change the pattern of serous papillary architecture; however, the number and types of tumor-infiltrating immune cells may be varying and should be further analyzed.

Active substances or molecules that bind and activate TLRs are one of the main focuses of cancer management, including for OC [11, 28, 29]. In this regard, we showed that P-MAPA promotes significant antitumor activity and potentiates the effects of CIS upon TLR signaling. Notably, P-MAPA upregulates TLR2 and TLR4 in OC, and seems to produce an additive effect on TLR4 expression in the presence of CIS. It is undisputed that TLR activation leads to the development of adaptative immune response by stimulating gene expression related to inflammatory mediators [13, 30, 31]. Furthermore, the prosurvival and proinflammatory actions of TLR signaling in cancer and immune cells may drastically influence tumor progression. Because the OC microenvironment is particularly immunosuppressive, therapies continue to stimulate the effective immune response against tumor cells [32]. In immune cells, TLR2 and TLR4 can induce the antitumor activities by enhancing the function of antigen-presenting cells (APCs), and further, the activation of lymphocyte subsets (especially cytotoxic T cells and NK cells). A number of clinical trials have been conducted to stimulate TLRs in OC therapy, such as TLR agonist in combination with dendritic cells (DC) vaccines [33, 34]. Thus, TLR agonist-based immunotherapies (e.g. P-MAPA) may also be successful for the treatment of serous OC.

In addition to TLR4 activation, P-MAPA and CIS upregulated downstream molecules such as MyD88, TRIF, p-IkBα, and NF-kB p65. An animal in vitro study reported that CIS treatment enhanced TLR4 expression and induced the phosphorylation of IkB in macrophages, making them functionally more responsive to TLR ligands [20]. In our study, P-MAPA had an additive effect on CIS-induced TLR4 activation. Furthermore, our results showed that P-MAPA in association with CIS might act to induce the expression of TLR4-mediated inflammatory genes via MyD88- and TRIF-dependent signaling pathways. In fact, P-MAPA therapy is thought to exacerbate the downstream activation of these pathways to activate NF-kB p65 [15]. Due to its agonist properties, P-MAPA may be considered as an additional therapy by activating immune cells in OC.

TLR4/MyD88/NF-kB signaling is found in OC cells and significantly influences drug response [5]. Undoubtedly, NF-kB plays a pivotal role in the TLR4-mediated signaling in cancer cells. Under normal conditions, NF-kB is retained in the cytosol by binding to an inhibitory protein IkB. Activated IKKs lead to phosphorylation of IkB, and the NF-kB p65 is translocated to the nucleus [35]. The mechanisms underlying resistance to chemotherapy and long-term cell survival in serous OC are directly associated with NF-kB pathway [36]. This study is the first to demonstrate that the combination of P-MAPA with CIS significantly increased both cytoplasmic and nuclear p65, thus promoting the high expression and activity of p65. The nuclear localization of NF-kB, a key event for NF-kB transactivation, is differentially altered by a number of drug treatments. According to Gaikwad et al. [37], the lowest levels of nuclear NF-kB were observed after treatment of ovarian A2780 cells with CIS, whereas an increased expression of NF-kB was evident in paclitaxel-treated cells. These effects indicate that activation of NF-kB is important for the maintenance of CIS resistance after silencing the MyD88 adapter molecule. Because CIS alone increased the expression of MyD88, p-IkBα, IKK-α, and nuclear factor p65, and further showed a profound reduction in OC volume and mass, we believe that CIS treatment was unable to promote chemoresistance in these serous OC cells derived from a chemically induced rat model.

Notably, OC has shown an increased resistance to paclitaxel, a TLR4 ligand, but not to platinum derivatives [3]. Regarding different OC cell lines (OVCAR3, SKOV3, A2780), the TLR4 activation induced IL-1 receptor-associated kinase (IRAK)-4 and NF-kB signaling, and c-Jun, IL-6 and IL-8 production, all of which related to tumor chemoresistance [5]. Although the activation of TLR system appears to be associated with poor outcomes when considering OC cells, our results suggest a protective role of TLR4 signaling in relation to the management of immune cells.

We verified activation of the TLR system in addition to nuclear translocation of NF-kB in OC cells; however, the elevation in TNF-α and IL-6 levels was insignificant following the treatments. Novel experiments involving different periods and regimens could increase this response or even indicate molecular alterations during the final production of these cytokines. Although the effects of P-MAPA and P-MAPA + CIS were insufficient to enhance the levels of IFN-γ, there was a biologically relevant potential of P-MAPA in stimulating the innate immune system in OC, especially eliciting a polarized Th1-type response with high production of IFN-γ. These findings were recently confirmed by Garcia et al. [14, 15], who concluded that P-MAPA was responsible for recovering the immunosuppressive microenvironment of bladder cancer than other conventional therapies. In bladder cancer, intravesical P-MAPA immunotherapy promoted a distinct activation of the TLR2- and TLR4-mediated innate immune system, resulting in increased IFN-γ signaling (TRIF-dependent pathway), which was more effective in the treatment of this tumor [15]. Furthermore, activation of IFN signaling pathway induced by P-MAPA upregulated the inducible nitric oxide synthase (iNOS) and wild-type p53 protein, resulting in apoptosis and histopathological recovery of the bladder cancer [15]. Another important therapeutic approach for bladder cancer was demonstrated by Dias et al. [38], based on association of P-MAPA with systemic administration of CIS, being much more effective, well tolerated, and with no apparent signs of drug antagonism. Whether P-MAPA regulates the cellular metabolism is of interest since this model of serous OC shows significant changes in diverse metabolic pathways [39, 40].

More importantly, the success of novel mechanistic insights into tumor immunotherapy is hindered by various obstacles including the ability of OC cells to generate an immunotolerant environment, the activation of negative regulatory signals, and the secretion of inhibitory factors and immunosuppressive cytokines. Lastly, studies exploring the effect of P-MAPA and CIS on genetic TLR2 and TLR4 knockout mouse models of OC are needed to better understand the molecular mechanism(s) by which these therapies effectively promote their immunomodulatory effects in OC.

## Conclusion

In summary, our study demonstrated the cross-talk between P-MAPA and CIS upon TLR system. Although CIS therapy was more effective than P-MAPA in reducing tumor growth, P-MAPA immunotherapy significantly increased the expressions of TLR2 and TLR4. Together, P-MAPA and CIS therapy is important in modulating the downstream molecules of MyD88- and TRIF-mediated TLR4 signaling pathway in OC. While no apparent effect on proinflammatory cytokines was observed, P-MAPA alone or in combination with CIS seems to cause elevation in IFN signaling. Our data suggest that P-MAPA immunotherapy may provide additional therapeutic opportunity for OC in combination with other anticancer agents, such as cisplatin. This may allow the discovery of new immune mechanisms during the acquisition of chemoresistance to drugs.

## Abbreviations

ANOVA: Analysis of variance
APC: Antigen-presenting cells
BSA: Bovine serum albumin
CEEA: Ethical Committee of the Institute of Bioscience/UNESP
CEMIB: Multidisciplinary Center for Biological Investigation
CIS: Cis-Diamminedichloroplatinum (Cisplatin)
DAB: Diaminobenzidine
DAPI: 6-diamidino-2-phenylindole
DC: Dendritic cells
DMBA: 7,12-dimethylbenz(a)anthracene
ELISA: Enzyme-linked immunosorbent assay
FITC: Fluorescein Isothiocyanate
H&E: Hematoxylin and Eosin staining
HRP-conjugated: Horseradish peroxidase-conjugated
IFN: Interferon
IFN-γ: Interferon gamma
IHC: Immunohistochemistry
IkB: I kappa B kinase
IKK-α: I kappa B kinase alpha
IL-6: Interleukin 6
IL-8: Interleukin 8
IRAK-4: Interleukin-1 receptor-associated kinase
IRF: Interferon regulatory factor
IRF3: Interferon regulatory factor 3
MAPK(s): Mitogen-activated protein kinase(s)
MyD88: Myeloid differentiation factor 88
NF-kB p65: Nuclear factor kappa B subunit p65
NK: Natural killer cells
OC: Ovarian cancer
PBS: Phosphate-buffered saline
p-IkBα: Inhibitor of phosphorylated NF-kB alpha
P-MAPA: Protein aggregate magnesium-ammonium phospholinoleate-palmitoleate anhydride
RIPA: Radioimmunoprecipitation assay buffer
RT: Room temperature
SD: Standard deviation
SDS: Sodium dodecyl sulphate
SDS-PAGE: Sodium dodecyl sulphate-polyacrylamide gel electrophoresis
TBS-T: Tris-Buffered Saline plus Tween 20
TCD4 +: T cells CD4-positive
TCD8 +: T cells CD8-positive
Th1: T helper 1
TLR(s): Toll-like receptor(s)
TLR2: Toll-like receptor 2
TLR4: Toll-like receptor 4
TNF-α: Tumor necrosis factor alpha
TRIF: TIR domain-containing adaptor inducing interferon-beta
